# Supplementary material for: Modeling glioblastoma heterogeneity as a dynamic network of cell states
Source: Mol Syst Biol. 2021 Sep 16;17(9):e10105. doi: 10.15252/msb.202010105 (PMC8444284; doi:10.15252/msb.202010105)
Supplement: Supplementary file 5 — Source Data for Figure 3 [file MSB-17-e10105-s001.zip › Figure3A_sourcedata/GSEA_3065/nosigdb_state3.GseaPreranked.1622718426028/SUVA_OPC_PMID_31327527.html]

Details for gene set SUVA\_OPC\_PMID\_31327527[GSEA]

|  || Dataset | state3 |
| Phenotype | NoPhenotypeAvailable |
| Upregulated in class | na\_pos |
| GeneSet | SUVA\_OPC\_PMID\_31327527 |
| Enrichment Score (ES) | 0.64831084 |
| Normalized Enrichment Score (NES) | 2.0693169 |
| Nominal p-value | 0.0 |
| FDR q-value | 4.59561E-4 |
| FWER p-Value | 0.003 |
Table: GSEA Results Summary

  

Fig 1: Enrichment plot: SUVA\_OPC\_PMID\_31327527      
 Profile of the Running ES Score & Positions of GeneSet Members on the Rank Ordered List

  

| PROBE | GENE SYMBOL | GENE\_TITLE | RANK IN GENE LIST | RANK METRIC SCORE | RUNNING ES | CORE ENRICHMENT || 1 | BCAN |  |  | 0 | 0.883 | 0.1052 | Yes |
| 2 | SCRG1 |  |  | 3 | 0.645 | 0.1819 | Yes |
| 3 | PTPRZ1 |  |  | 14 | 0.532 | 0.2442 | Yes |
| 4 | PGRMC1 |  |  | 29 | 0.473 | 0.2990 | Yes |
| 5 | ALCAM |  |  | 45 | 0.440 | 0.3497 | Yes |
| 6 | GPM6B |  |  | 105 | 0.343 | 0.3839 | Yes |
| 7 | TTYH1 |  |  | 183 | 0.293 | 0.4099 | Yes |
| 8 | PCDHGC3 |  |  | 228 | 0.272 | 0.4373 | Yes |
| 9 | SERINC5 |  |  | 252 | 0.263 | 0.4660 | Yes |
| 10 | VCAN |  |  | 293 | 0.245 | 0.4907 | Yes |
| 11 | THY1 |  |  | 326 | 0.236 | 0.5152 | Yes |
| 12 | CADM2 |  |  | 416 | 0.215 | 0.5306 | Yes |
| 13 | NLGN3 |  |  | 558 | 0.191 | 0.5372 | Yes |
| 14 | EPB41L2 |  |  | 596 | 0.187 | 0.5552 | Yes |
| 15 | OLIG1 |  |  | 602 | 0.186 | 0.5768 | Yes |
| 16 | SCD5 |  |  | 619 | 0.184 | 0.5969 | Yes |
| 17 | RNF13 |  |  | 635 | 0.181 | 0.6167 | Yes |
| 18 | LIMA1 |  |  | 639 | 0.180 | 0.6379 | Yes |
| 19 | P2RX7 |  |  | 723 | 0.167 | 0.6483 | Yes |
| 20 | APOD |  |  | 1113 | 0.129 | 0.6190 | No |
| 21 | TMEM206 |  |  | 1477 | 0.105 | 0.5898 | No |
| 22 | PLP1 |  |  | 2047 | 0.074 | 0.5334 | No |
| 23 | TNS3 |  |  | 3372 | 0.031 | 0.3852 | No |
| 24 | LMF1 |  |  | 4056 | 0.014 | 0.3085 | No |
| 25 | FIBIN |  |  | 4788 | -0.006 | 0.2253 | No |
| 26 | SIRT2 |  |  | 4877 | -0.008 | 0.2161 | No |
| 27 | PHYHIPL |  |  | 4985 | -0.010 | 0.2051 | No |
| 28 | FGF12 |  |  | 5669 | -0.029 | 0.1302 | No |
| 29 | CNTN1 |  |  | 5924 | -0.037 | 0.1054 | No |
| 30 | RTKN |  |  | 6452 | -0.055 | 0.0514 | No |
| 31 | FXYD6 |  |  | 6822 | -0.072 | 0.0177 | No |
| 32 | PSAT1 |  |  | 7634 | -0.131 | -0.0597 | No |
| 33 | CNP |  |  | 7852 | -0.158 | -0.0658 | No |
| 34 | DBI |  |  | 8571 | -0.383 | -0.1025 | No |
| 35 | RAB31 |  |  | 8696 | -0.514 | -0.0555 | No |
| 36 | FABP5 |  |  | 8699 | -0.518 | 0.0060 | No |
Table: GSEA details [plain text format]

  

Fig 2: SUVA\_OPC\_PMID\_31327527: Random ES distribution      
 Gene set null distribution of ES for **SUVA\_OPC\_PMID\_31327527**
